# Bronchus-associated lymphoid tissue lymphoma stage IV with subsequent histologic transformation to an aggressive lymphoma: A case report

**DOI:** 10.1186/1752-1947-5-455

**Published:** 2011-09-12

**Authors:** Rajeev Swarup

**Affiliations:** 1 Division of Pulmonary and Critical Care Medicine, Henry Ford Hospital, Detroit, MI 48202, USA

## Abstract

**Introduction:**

Extranodal marginal B-cell lymphoma of bronchus-associated lymphoid tissue is a rare entity accounting for less than 1% of all lymphomas. We report a case of a low-grade bronchus-associated lymphoid tissue lymphoma stage IV with transformation to an aggressive large B-cell lymphoma.

**Case presentation:**

A 59-year-old African-American man was incidentally found to have a bronchus-associated lymphoid tissue lymphoma involving the bilateral lower lobes of his lungs. In addition, bone marrow involvement was discovered. His course was indolent with only some mild respiratory symptoms. He received single agent treatment with rituximab. No evidence of progression was seen at the end of receiving this regimen. Two years after treatment our patient presented with B symptoms. Imaging now showed significant increase in the size of the lung masses with cavitation of the right lower lobe mass. A repeat transbronchial biopsy suggested transformation to an aggressive diffuse large B-cell lymphoma.

**Conclusion:**

This case illustrates a rare bronchus-associated lymphoid tissue lymphoma stage IV with histologic transformation to an aggressive lymphoma. In addition, this rare case of transformation presented as a cavitary lesion.

## Introduction

Primary pulmonary non-Hodgkin's lymphoma or lymphoma of bronchus-associated lymphoid tissue (BALT) is a rare entity accounting for less than 1% of all lymphomas [1]. This malignancy is characterized by an often indolent course, in patients that are often asymptomatic, and is frequently found incidentally on chest radiography. In most cases, BALT lymphoma is localized to one lung but can involve both lungs [2-4]. In a few cases the bone marrow is involved [5]. Histologic transformation has been described in other types of low-grade lymphoma including follicular and gastric lymphomas [6]. This report represents an extremely rare case of stage IV BALT lymphoma with histologic transformation to an aggressive diffuse large B-cell lymphoma.

## Case Presentation

**Figure 1:**
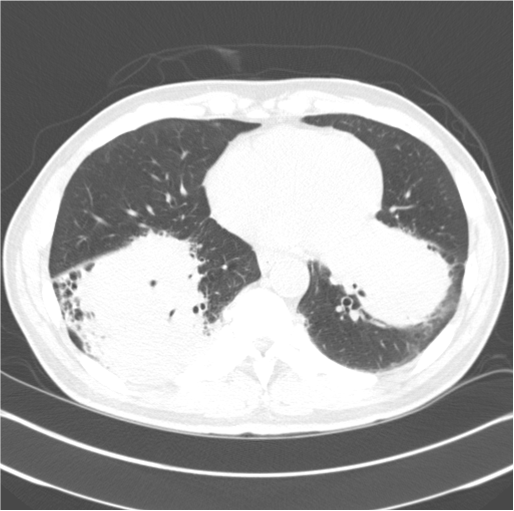
**Initial CT scan performed in 2008**. CT of thorax without contrast, showing bilateral mass-like opacities.

**Figure 2:**
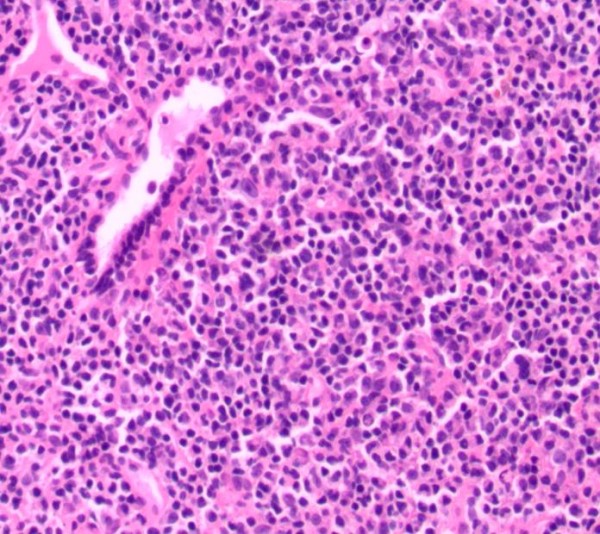
**Initial histology of pulmonary lesion in 2008**. Slide showing lymphocytes with plasmacytoid features--uniform, small, and in abundance; no air spaces seen (hematoxylin and eosin stain; magnification 20×).

**Figure 3:**
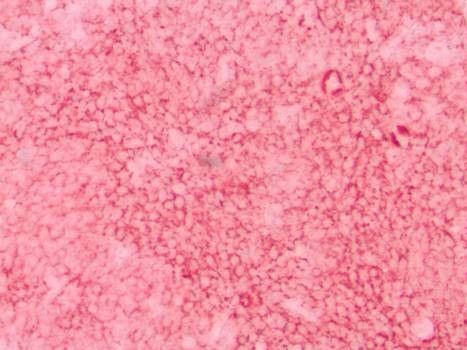
**Histology of bone marrow biopsy**. L-26 B-cell stain showing an abundance of B-cells. Flow cytometry of bone marrow with CD19+, CD20+, CD5-, kappa+ monoclonal B-cells consistent with low-grade B-cell lymphoma.

A 59-year-old African-American man, with history of renal insufficiency, diabetes, hypertension, severe peripheral vascular occlusive disease, and a 100 pack-year tobacco history, was incidentally found to have an abnormal chest radiograph showing bilateral large lower lobe airspace opacities. Images taken three years previously were reviewed; the abnormalities were present at that time and had not changed in size significantly. The patient was lost to follow-up after that initial abnormal radiograph. He was experiencing only mild respiratory complaints of dyspnea on exertion and occasional dry cough. There were initially no symptoms of hemoptysis, fever, chills, weight loss or night sweats. No laboratory abnormalities were discovered. A history of diagnosed autoimmune disease, cancer or family history of lung disease was not present. A computed tomography (CT) scan of his chest confirmed dense consolidation with air bronchograms involving several segments of his bilateral lower lobes. No mediastinal lymphadenopathy was seen (see Figure 1). Our patient underwent bronchoscopy with a transbronchial biopsy. The pathology of the biopsy specimen showed extranodal marginal zone B-cell lymphoma of BALT (see Figure 2 and 3). Further staging with positron emission tomography-CT and bone marrow biopsy revealed stage IV disease involving the subcarinal lymph nodes as well as the bone marrow.

**Figure 4:**
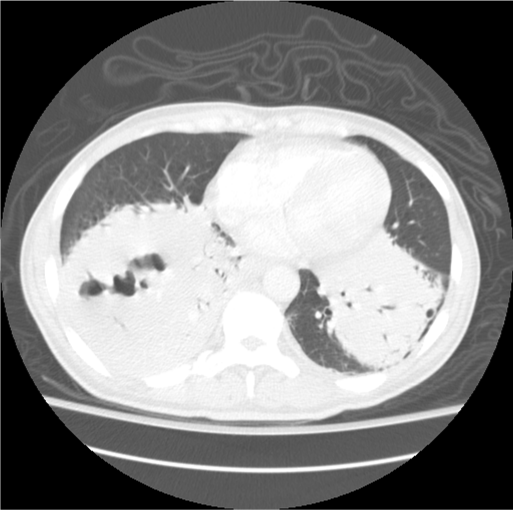
**Subsequent CT scan performed in April 2008**. CT of thorax showing cavitation of right lower lobe previously low-grade BALT lymphoma.

Our patient was treated with single-agent rituximab and received four total doses, one dose given weekly. His clinical course remained stable with no evidence of progression and some improvement on CT imaging post-treatment. Our patient was seen every three months by his oncologist after his initial diagnosis. Two years after the diagnosis and treatment with rituximab our patient's clinical course worsened. He described new symptoms of worsening shortness of breath, weight loss, night sweats and fevers. He had elevated lactate dehydrogenase at 350IU/L, uric acid measuring 10.0 mg/dL and creatinine of 1.7 mg/dL. A repeat CT scan showed new air-filled areas within the right lower lobe mass, consistent with cavitations representing probable necrosis (see Figure 4). A comprehensive work-up for infection was negative, which included bacterial and fungal testing, studies for acid-fast bacilli and human immunodeficiency virus testing.

**Figure 5:**
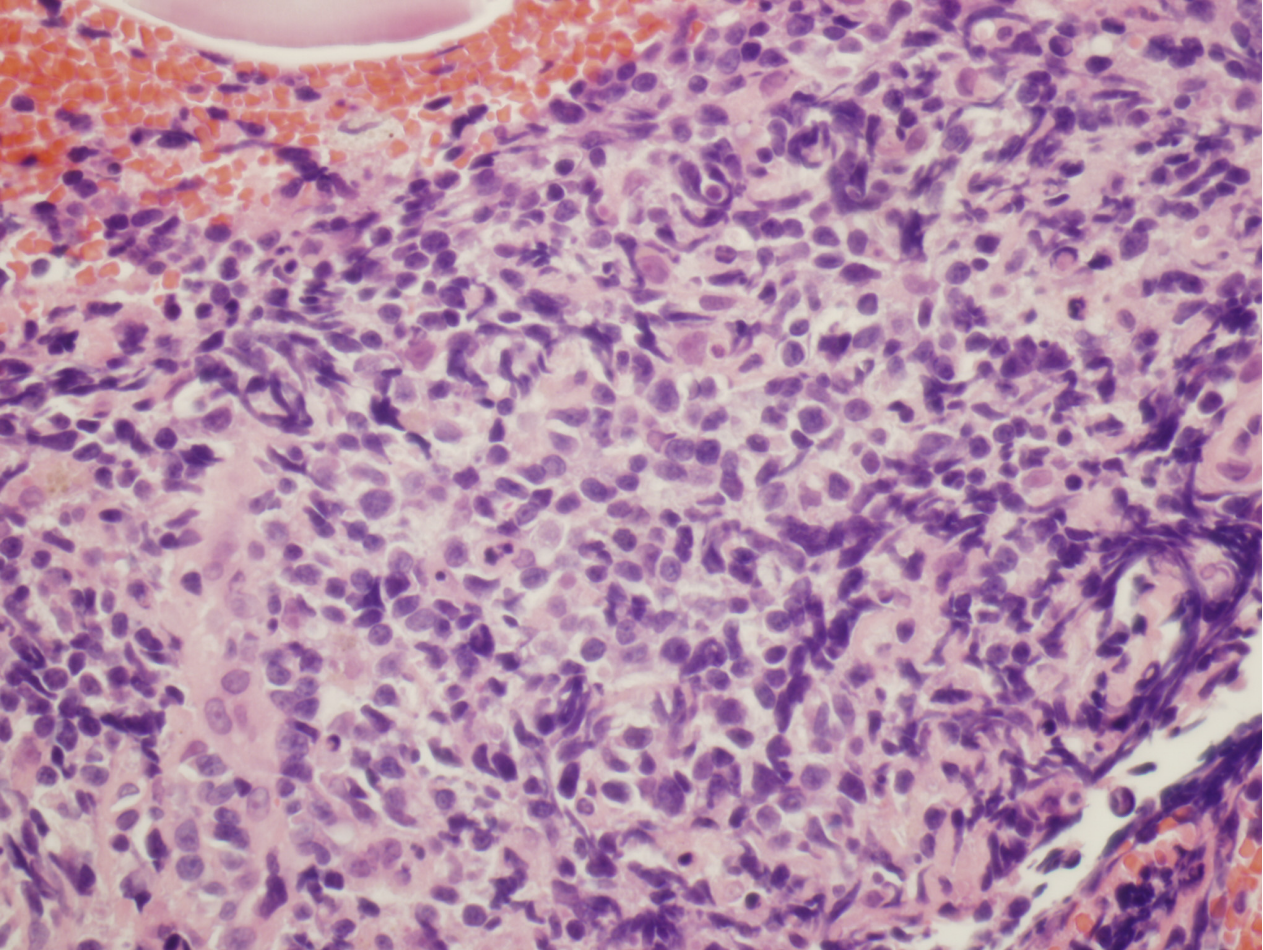
**Histology of pulmonary lesion on repeat biopsy in 2011**. There is an increased large cell population, and the proliferation index is elevated, suggesting transformation to diffuse large B-cell lymphoma (hematoxylin and eosin stain; magnification 60×).

Clinical concern for transformation of this low-grade lymphoma to a more aggressive form was also considered. Our patient underwent a repeat transbronchial biopsy which suggested transformation to a diffuse large B-cell lymphoma secondary to findings of an increased large cell population (see Figure 5). Due to these new findings our patient was started on the chemotherapy regimen of rituximab-CHOP. He completed two cycles and five days later was admitted to the hospital for hyperglycemia. His hospital course became complicated by the development of a new loculated pneumothorax involving his right lung, thought to be secondary to the necrotic lung process. He required chest tube placement and was placed on mechanical ventilation. His condition worsened with development of severe sepsis secondary to *Enterococcus faecium *in sputum, pleural fluid and blood.

Due to multiorgan system failure and poor overall prognosis the family changed the goal of care to palliative and our patient died shortly thereafter.

## Discussion

Extranodal marginal B-cell lymphoma of BALT is an extremely rare form of primary lung lymphoma. From reviews of reported cases, bone marrow involvement occurs even less frequently [4]. Case series have shown that the natural history of this malignancy is often indolent, as was the case initially in our patient. Up to one-third of patients are asymptomatic at presentation and most patients have disease localized to one lung. In some cases both lungs are involved. The radiographic abnormalities seen are variable and represent lung nodules, airspace consolidation, or less seen mass lesions [1-4,7-9]. The five-year survival in patients with BALT lymphoma is favorable and reported to be 85% [5].

These low-grade malignancies have been associated with chronic local inflammation states, such as those seen in chronic hypersensitivity pneumonitis and smoking. Autoimmune diseases, including Sjögren's and rheumatoid arthritis, have also been associated with development of these primary lymphomas [9-12]. The optimal management of BALT lymphoma has not been clearly defined and currently involves surgery, chemotherapy, radiotherapy and abstention in some cases [4,9]. Our patient was treated initially with rituximab monotherapy, which appeared to halt progression for two years. The use of rituximab in treatment of BALT lymphoma is reported in several case series and is based on the effects the drug on CD20 antigens present on the surface of BALT lymphoma [1,4]. No randomized control trials have been done to show efficacy in treatment of this rare malignancy.

Clinical deterioration was seen in our patient after two years with new findings of B symptoms and radiographic changes showing cavitation of right lower lobe malignancy. A transbronchial biopsy suggested transformation to more aggressive large B-cell lymphoma, supporting the clinical picture of histogical transformation suggested by the presence of the B symptoms. In our review of the literature, transformation of low-grade indolent B-cell lymphomas is described and most commonly seen in follicular lymphoma transforming to large B-cell lymphoma. This transformation has also been reported in gastric mucosa-associated lymphoid tissue [6]. To the best of our knowledge, although reported in gastric mucosal-associated lymphoid tissue, transformation of BALT lymphoma to large B-cell lymphoma is extremely rare. In addition, cavitation of the transformed large B-cell lymphoma found in our patient is only reported in a few case reports [13-15].

## Conclusion

We have presented a rare case of BALT lymphoma stage IV. Our patient was initially treated with rituximab and had stabilization of his disease for two years. He deteriorated clinically with development of B symptoms and his initial cancer was found to have transformed to an aggressive cavitary large B-cell lymphoma.

## Abbreviations

BALT: bronchus-associated lymphoid tissue; CHOP: cyclophosphamide, hydroxydaunorubicin, oncovin, prednisone; CT: computed tomography

## Consent

Written informed consent was obtained from the patient's next of kin for publication of this case report and any accompanying images. A copy of the written consent is available for review by the Editor-in-Chief of this journal.

## Competing interests

The authors declare that they have no competing interests.
